# Anthropometrics, body composition, sarcopenia, and frailty in intestinal transplantation: A literature review and commentary

**DOI:** 10.1016/j.intf.2024.100020

**Published:** 2024-10-16

**Authors:** Faruq Pradhan, Yara Dababneh, Thomas Pietrowsky, Syed-Mohammed Jafri, Shaheed Merani

**Affiliations:** a Division of Gastroenterology and Hepatology, Department of Internal Medicine, University of Nebraska Medical Center, Omaha, NE 68198, USA; b Division of Gastroenterology and Hepatology, Department of Internal Medicine, Henry Ford Hospital, Detroit, MI, 48202, USA; c Solid Organ Transplant and Intestinal Rehabilitation, Henry Ford Hospital, Detroit, MI 48202, USA; d Division of Transplant, Department of Surgery, University of Nebraska Medical Center, Omaha, NE 68198, USA

**Keywords:** Organ transplantation, Sarcopenia, Frailty, Short bowel syndrome, Intestinal failure

## Abstract

Frailty and sarcopenia are increasingly incorporated in the evaluation of solid organ transplant candidates. There can be adverse outcomes both pre- and post-transplant amongst patients identified as frail. Nutritional deficiencies and chronic illness in intestinal transplantation are known to impact growth and muscle development. As a result, anthropometrics and body composition have been studied amongst patients with intestinal failure. More recently, sarcopenia and frailty are phenotypes that have been identified as independent factors that can impact morbidity and mortality among patients undergoing solid organ transplantation. It is worthwhile to review available knowledge regarding frailty and sarcopenia in both adult and pediatric patients undergoing intestinal transplantation evaluation. This review article summarizes existing data regarding anthropometrics, sarcopenia, and frailty in adult and pediatric patients under consideration for intestinal transplantation. An opinion statement is provided discussing potential means of assessing frailty in this population, along with recommendations in the pre- and post-transplant phases of care.

## Background

Nutritional deficiencies and chronic illness in intestinal transplantation are known to impact growth and muscle development. As a result, anthropometrics and body composition have been studied amongst patients with intestinal failure. Sarcopenia and frailty are phenotypes that have been identified as independent factors that can impact morbidity and mortality among patients undergoing solid organ transplantation [1]. An increased risk of adverse post-transplant outcomes is observed in patients with pre-existing frailty. Much less data, however, is available for patients undergoing IT compared to other solid organ transplants. We aimed to investigate the current literature surrounding the role of anthropometric, body composition, sarcopenia and frailty measurements in both pediatric and adult intestinal transplantation candidates and recipients.

Intestinal failure is a condition resulting in malnutrition, which arises from a variety of etiologies, including short gut syndrome. Current diagnoses requiring intestinal transplantation consideration include necrotizing enterocolitis, congenital short gut syndrome, intestinal pseudo-obstruction, or other similar enteropathies [2]. Malnutrition from the disease process or complications resulting from total parenteral nutrition (PN), is associated with high morbidity and mortality in both pediatric and adult patients with the condition [3], [4]. Complications of PN include factors which may further impact sarcopenia including cholestatic liver disease, sepsis from central line-associated bloodstream infections, renal dysfunction, metabolic bone disease, and venous thrombosis [4]. Recent advances in the management of intestinal failure include specific formulations of PN which have reduced complications such as cholestatic liver disease and the use of fish oil or multi-oil formulations of lipids [5], [6], [7], multidisciplinary intestinal rehabilitation programs [4], [8], [9], and the use of growth hormone therapy which can reduce or eliminate the need for PN [10], [11]. Improved management of central venous catheters, including ethanol lock therapy [12], has also been demonstrated to be beneficial in reducing catheter-related bloodstream infections, which reduces rates of sepsis but also decreases the need for venous manipulation, thereby reducing the risk of thrombosis. Refractory advanced liver disease, recurrent episodes of sepsis, or loss of two or more central venous sites due to thrombosis merit intestinal transplant consideration (whether in isolation or in combination with liver transplantation). In the United States, there has been a continued demand for intestinal transplants across adult and pediatric candidates [2] over the past ten years, with over 40 % of patients remaining on the list for over two years.

## Anthropometrics and body composition

Anthropometrics are an essential aspect of nutritional assessment, especially in pediatric populations. Weight, height, length, head circumference, mid-arm muscle circumference, and skin fold thickness are measurements often used for comparing patients with intestinal failure against the general population and for monitoring nutritional intervention outcomes [13]. These measurements, however, fail to incorporate more detailed assessments of body composition, such as fat-free mass index, fat mass index, and bone mineral density. Patients with short bowel syndrome who have normal linear growth or body-mass index can mask muscle mass deficiency [14] and would merit a different nutritional strategy (such as increased amino acid/protein needs) compared to patients with normal body composition along with physical therapy or a structured exercise program [15]. Therefore, anthropometry in isolation may be insufficient to evaluate overall health, nutrition, prognosis, or outcome and ultimately for quality of life, especially in short bowel syndrome or post-IT.

Measures of body composition including muscle mass and bone mineral density are useful but do not require specialized equipment for measurement. Other measures of body composition such as dual energy X-ray absorptiometry (DEXA) and air displacement plethysmography (ADP) are often not accessible to many patients or clinicians. Bioelectrical impedance analysis (BIA) is a non-invasive and relatively low-cost method of quickly determining body composition. Depending on the sensors used for BIA, this technology can predict fat mass, fat free mass, and body fluids composition [16].

## Sarcopenia and frailty

Measuring sarcopenia in post-intestine transplant patients can help assess improved intestinal absorption of nutrients. Sarcopenia describes the evaluation of not only loss of muscle mass but also functional impairment and decreased strength [17]. Three criteria are utilized: low muscle strength, low muscle quantity or quality, and low physical performance. Severe sarcopenia is diagnosed if all three criteria are identified in a patient [18]. Though sarcopenia was initially investigated in geriatric patients, there has been growing research in understanding the prevalence of sarcopenia in children, along with outcomes surrounding growth and neurocognitive development [19]. The presence of sarcopenia in non-intestinal adult solid organ transplant recipients is associated with an increased risk of post-transplant peri-operative complications, poor graft survival, and an overall increased risk of death [20]. Limited data exists for intestinal transplant however, in one study, the prevalence of sarcopenia in pediatric intestinal transplant recipients was estimated to be 64 %, sarcopenia was not associated with worse outcomes [21].

There are a multitude of tools developed to screen and diagnose sarcopenia that have been utilized both in intestine transplant and non-transplant populations (both pediatric and adult). Examples of tests available are summarized in Table 1, including data on pediatric and adult patients, and whether these tools have been used in an intestinal failure or small bowel transplant population. Given the dynamic fluid changes often seen in these patients related to TPN, testing that is often utilized in other populations (such as whole-body DEXA) can be less effective in intestinal failure patients or in the peri-transplant period. As such, the nutrition-focused physical examination is of vital importance in the assessment of these patients. Adipose tissue measurements as a part of the nutrition-focused physical examination have also been investigated in both adult and pediatric IT recipients and have been found to correlate against sarcopenia (as identified on CT skeletal muscle index measurements) [22], though, in this single-center study, pre-IT malnutrition or sarcopenia did not predict post-transplant infections, length of stay, nor mortality. This is a notable finding and highlights the need for further study in this special population, as these findings are not congruent with non-intestinal solid organ transplant recipients with pre-transplant sarcopenia.

**Table 1:** Sarcopenia assessment techniques.

Technique	Adult, Pediatric, Both	Intestinal failure or transplant data available	Advantages	Disadvantages	References
SARC-F Questionnaire	Adult	No	- Minimal equipment and training necessary - Highest sensitivity for severe sarcopenia - Portable - No cost to preform	- Includes subjective parameters - Does not provide specific fat/tissue mass data	[18], [46]
CT – Skeletal Muscle Index	Both	Limited	- Objective measure - Unaffected by volume status	- Costly - Racial background may affect accuracy - Need for radiation - Need for specialized software and/or expertise - Limited cross-sectional area investigated	[22], [47], [48]
MRI	Adult	No	- Objective measure - Unaffected by volume status - Lack of radiation - Ability to evaluate for muscle quality (including myosteatosis)	- Costly - Need for specialized software and/or expertise - Limited cross-sectional area investigated	[49]
Whole-body DEXA	Both	No	- Includes bone density evaluation - Includes fat and lean tissue mass data - Ability to evaluate whole-body data	- Can be affected by changes in volume status - Fat and lean tissue masses are not routinely reported on standard DEXA - May require sedation for children under 2 years old - Requires radiation	[50], [51]
Bioelectric impedance analysis	Both	Adult-only	− Portable − Cost-effective − Can provide muscle and fat quantification data − No radiation exposure	- Reference range varies based on equipment, ethnic background	[14], [50]
Air Displacement Plethysmography	Pediatric	Yes	− No radiation exposure − Reference levels are available for infants − Can evaluate fat and fat-free mass	− Can only provide whole-body data − Expensive device	[15]

Frailty describes a state of decreased physiological reserve in addition to an increased vulnerability to stressors that can lead to repeat hospitalization, impaired quality of life, and death [1]. Though sarcopenia can often be seen in this population, frailty describes the clinical phenotype and incorporates a patient’s nutritional status [23]. The identification of frailty prior to (non-intestinal) solid-organ transplant has been of rapidly growing importance, given the risks to mortality, length of stay, postoperative complication, allograft function, and waitlist mortality [1]. There is a lack of knowledge about frailty in the intestinal transplant population, however. In the general medical population, popular measures of frailty include the Clinical Frailty Score [24], FRAIL [25], Fried’s Frailty phenotype [26], and the Short Performance Physical Battery [27]. Many of these indices are subjective based on clinician or patient-reported functional status, while others combine subjective factors along with objective measures such as hand grip strength or ability and time required to perform certain sitting and standing tasks. For adult patients with cirrhosis, the Liver Frailty Index [28] has been developed as an objective tool to identify patients with impaired muscle strength and low physical performance. This has also been validated in adult liver transplantation in both pre-transplant [28] and post-transplant [29] settings, with frail patients demonstrating significant morbidity, increased waitlist mortality, and increased healthcare utilization in patients identified as pre-frail or frail [30]. Currently, there is no similar tool specific only to patients undergoing intestinal transplantation (either pediatric or adult).

## Pediatric intestinal transplant

### Candidate evaluation for intestinal transplant

During the evaluation of a potential intestinal transplant candidate, a multidisciplinary team evaluation including medical and surgical risks and benefits is performed. This includes nutrition, functional, and psycho-social considerations. In a study from 2017, the intestinal group in Indiana described concurrently evaluated sarcopenia and fat stores in pediatric liver, kidney, and intestinal failure patients, demonstrated that intestinal failure patients tended to have closer to normal total protein levels and BMI compared to controls, however worse sarcopenia index (i.e., loss of lean muscle mass) and a concurrent increase in visceral fat index. This observation supports the concept of sarcopenic obesity [31], a condition in where BMI may be abnormally elevated even in a patient who is nutritionally and functionally deplete. The authors concluded that sarcopenia results in physical weakness, and loss of physical stability and stamina. Furthermore, the authors hypothesized that reliably diagnosing the presence of sarcopenia and describing its severity can assist clinical care. Within this study the presence of sarcopenic obesity as an entity in pediatric intestinal transplant candidates was confirmed – as these children lose muscle mass, they simultaneously increase fat stores and maintain their weight and BMI.

### Early post-transplant outcome

Following the initial description of improved patient survival with intestinal transplant versus ongoing intestinal rehabilitation in select patients with intestinal failure, there have been several groups investigating the longer-term outcomes, specifically the nutrition and physical outcomes of the pediatric intestinal transplant. Nucci et al. investigated the concept of catch-up growth, evaluated by weight and linear growth (i.e., height), using the Z-score to compare an individual's growth trajectory to the expected age-adjusted weight and height [32] following IT and cessation of PN. However, with a limited sample size and the lack of control for patient age groups, the correction for important factors such as age-dependent capacities for catch-up growth was not clear. What was apparent was that even out to 2 years following transplant, the Z-scores for weight and height were low, steady, and remained below zero. Nucci et al. therefore concluded that positive linear growth remained difficult post-IT despite liberation from parenteral nutrition and high survival rates. The authors postulated that steroids for immunosuppression as well as management of rejection was negatively affecting growth [32]. The authors further noted no differences between isolated intestinal and MVT recipients. Similar findings were concurrently reported from Nebraska by Iyer et al. in a cohort of 47 pediatric intestinal transplant recipients. Iyer et al. noted a lack of catch-up growth even at 2-years following transplant concluding that a majority of patients demonstrated a significant delay of linear growth at time of transplant that persisted up to two years post-IT. In this cohort, body weight after transplantation showed little catch-up growth, and instead continues at the same velocity with the standard deviation (z-score) being maintained. [33].

About a half a decade after this observation, Ueno et al. reported post-pediatric IT catch-up weight and height being observed as early as 6 months especially in patients with pretransplant Z-score below −2.0 [34]. This finding may have been related to the fact that the median age at transplant was 1 year in this study or due a difference in steroid protocol, whereas the median age of transplant in preceding studies was older (3.4 years vs. 3.7 years).

In a Spanish cohort of 20 pediatric intestinal or intestine-liver transplant recipients from 2006, both weight and height showed catch-up growth concordant with increased albumin and total protein levels [35]. The results of this observation should be interpreted in the context of a selection bias resulting from the analysis of only patients who survived past 1-year post-transplant.

### Long term post-transplant outcome

The longest-term nutritional data relating to linear growth from the Paris group demonstrates that pediatric intestinal transplant recipients’ heights are lower at time of transplant and remains proportionally stagnant even up to 10 years post-transplant [36]. However, this may represent a cohort effect reflecting a greater reliance on steroids in immunosuppression protocols pre-2000 [37].

The earliest study to demonstrate true catch-up weight growth in the pediatric intestinal transplant group was from UCLA [38]. In this study group, patients were being transplanted at a median age of 3.7 years, however with a larger proportion of patients receiving liver containing allografts. Interestingly, this study concurrently reported serum pre-albumin which was lower in the pre-transplant group. This is likely reflecting the correlation of improved nutrition with catch up weight gain; however, linear growth remained delayed in this cohort.

Interval follow-up from the UCLA cohort which included a series of 50 pediatric intestinal transplant recipients became available in 2011 [39], showing persistent observation of catch-up weight gain following transplant. Factors associated with positive catch-up growth following intestinal transplant included female sex, short hospitalization duration and cessation of PN (surrogate marker of intestinal function), and fewer episodes of post-transplant enteritis. Additionally, steroid doses of < 0.1 mg/kg/d and the use of peptide-based formulas were associated with positive longer-term outcomes. This study also investigated longer-term biochemical markers of nutrition in the 4 years following transplant, noting that many of these micro-nutrient and vitamin deficiencies took up to 4 years to normalize. This was the first data to suggest that the previous focus on growth in pediatric patients during a 1–2 year interval post-transplant may have been too early to observe the dynamic changes in catch-up growth.

These same delays in catch-up growth are also seen in solid organ transplant patients with liver disease without underlying intestinal disease where chronic illness limits catch up growth, not typically observed until a minimum of 1 year following transplant and observed until 1 year following transplant with a plateau in catch-up growth at 18 months [40]. In kidney transplant recipients, catch-up growth is highly dependent on age at transplant, and can continue for 1 – 3 years following transplant in younger cohorts (especially if <5 years old). Disparate findings in previously reviewed single-center intestinal series may in part be explained by heterogeneity in demographic factors and immunosuppressive strategies rather than a true difference in the outcome of catch-up weight or linear growth following transplant [41].

More recently, the evaluation of functional and nutritional status in the form of frailty has shifted focus away from height and weight anthropometrics towards sarcopenia assessment. Total psoas muscle area has been recently investigated in pediatric intestinal transplant recipients [21]. Sarcopenia Z-score was low in the peri-transplant period and did not appear to improve significantly during the 5-years study follow-up period demonstrating that sarcopenia can persist despite catch-up weight growth. The most challenging finding to explain in this study [21] is the fact that the presence of sarcopenia was associated with higher rates of 1-year and 5-year patient survival although this could be the result of a Type I statistical error. The authors of this study hypothesized that sarcopenia may result in immunosuppression (possibly through decreased IL-6), and therefore lower rejection episodes in this population.

In the post-transplant setting, anthropometrics has been used in a pediatric population to evaluate the role of post-intestinal transplant adjunctive therapies – specifically, amongst pediatric IT recipients with growth delay receiving recombinant human growth hormone therapy [42]. Here height Z-score was used to demonstrate linear growth in the two years following initiation of recombinant growth hormone therapy with no serious adverse events or impact on allograft function noted.

## Adult intestinal transplant

In the adult setting, specialized rapid bed-side functional assessments including hand grip strength can be used [43] for sarcopenia assessment. Here, the focus was based on a comparison to pre-transplant or pre-morbid adult baseline rather than z-score. Interestingly at 1-year post-IT in this UK study, adult intestinal transplant recipients had stable to slightly lower weight, BMI and mid-arm circumference, but variable improvement in hand grip strength. In a series of 22 adult intestine and multi visceral patients from Pittsburgh [44], stable body weight, BMI, serum albumin, triceps skin-fold thickness, mid-arm muscle circumference was observed in the 12 months following transplant in a time interval that over 50 % of patients achieved nutritional autonomy.

Adult patients with pre-transplant malnutrition, as defined by nutrition focused physical exam had sarcopenia on CT scan [22] in a 2022 study by Dowhan et al. Although CT based sarcopenia measures were associated with clinical diagnosis of malnutrition, no difference in post-transplant short term complications was observed in this small cohort.

Overall, we find sparse literature regarding prevalence in adult patients with acquired short bowel syndrome and would merit further research/investigation.

## Discussion

Novel tools to evaluate sarcopenia are likely complementary to existing anthropometrics measures in both pediatric and adult intestinal transplant population. A comprehensive understanding of the intestinal transplant recipient’s nutritional and functional status should involve anthropometrics, frailty and sarcopenia assessment, physical examination, serum markers of nutrition, and evaluation of functional status.

With a myriad of sarcopenia and frailty assessments available, the challenge faced in the modern era is the validity of these measures and their role in prognostication, risk-stratification, and identification of patients at highest need for intervention. Questions that should be answered relating to any proposed measure should focus on how prognosis, diagnosis or treatment can and should be altered. Considering the resource availability and possible invasive nature of these measures in a patient experience that already involves a multitude of testing, treatment, and clinical evaluations, the risk-benefit ratio of all clinical measurements should be carefully considered.

At present, although the general perception is that measurement of sarcopenia and frailty are important, there are no standardized guidelines for their measurement nor interpretation especially amongst IT candidates. Nuances of the measures and patient population given heterogeneity within the intestinal transplant population need to be understood to fully interpret single center studies. Differences between the pediatric and adult population, presence of liver disease, along with etiology of intestinal failure should be considered. Furthermore, causality in the interplay between malnutrition in the setting of intestinal and liver failure with the ability to identify modifiable risk factors should be factored in future studies.

Therefore, we propose several recommendations regarding frailty and intestinal failure:

1) Both adult and pediatric patients undergoing workup for intestinal transplantation should be evaluated for sarcopenia and functional status.
2) Consider opportunities for pre-habilitation of patients listed for intestinal transplantation
3) Patients listed for intestinal transplantations should have repeat measures of sarcopenia
4) Sarcopenia and functional status should be evaluated following intestinal transplantation, and once normalized, on an annual basis as part of post-transplant follow-up
5) For patients with sarcopenia pre-transplant, the post-transplant phase of care should be modified with possible post-transplant nutritional supplementation or more intensive physical therapy
6) Patients who are identified to have progression in sarcopenia or worsening functional status should be closely monitored in combination with other factors including clinical history, nutrition status, anthropometrics, and physical examination should undergo clinically appropriate investigations to determine etiology and possibly modify nutritional interventions

What has been learnt from observational studies in the early era is that pediatric patients demonstrate minimal catch-up growth in the early period post-IT. More recent studies however show some degree of catch-up weight growth following pediatric intestinal transplant, with some even demonstrating linear growth years out after transplant. Differences between these observational studies likely relate to age at transplant, isolated small bowel vs liver-small bowel transplantation, immunosuppression (including steroid use), length of follow-up, bias from smaller select cohorts, and heterogeneity in post-operative care (nutrition and physical rehabilitation).

The development and validation of clinical tools to assess for frailty or sarcopenia (especially point-of-care testing) in patients with short bowel syndrome or post-IT will be of great clinical importance for this population. Recent studies have shifted attention from pure anthropometric measures towards sarcopenia assessment, which may be a superior measurement of nutritional status and physical conditioning/deconditioning. Authors would argue that ultimately quality of life and achieving realistic patient-oriented functional tasks is paramount [45].

Opportunities exist for prospective observational multicenter evaluation of anthropometrics and sarcopenia in intestinal transplant. The presence of sarcopenia may provide opportunities to improve patients' functional outcomes by identifying modifiable risk factors. Pre-transplant optimization would be ideal, but inherently is a challenging prospect due to the failure of PN being an indication for IT.

## Ethics statement

Ethics approval was not required for this review.

## Funding statement

This research did not receive any specific grant from funding agencies in the public, commercial or not-for-profit sectors.

## No acknowledgements

Not applicable.

## Author disclosures

Syed-Mohammed Jafri: Speaker for Abbvie, Gilead, Takeda. Advisory Board for Takeda, Ironwood.

Faruq Pradhan: Advisory Board for Madrigal Pharmaceuticals.

The other authors have no financial disclosures.

This research did not receive any specific grant from funding agencies in the public, commercial or not-for-profit sectors.

## CRediT authorship contribution statement

**Faruq Pradhan:** Writing – review & editing, Writing – original draft, Conceptualization. **Yara Dababneh:** Writing – review & editing, Writing – original draft. **Thomas Pietrowsky:** Writing – review & editing, Writing – original draft, Conceptualization. **Syed-Mohammed Jafri:** Writing – review & editing, Writing – original draft, Conceptualization. **Shaheed Merani:** Writing – review & editing, Writing – original draft, Supervision, Conceptualization.

## Declaration of Competing Interest

The authors declare the following financial interests/personal relationships which may be considered as potential competing interests Syed-Mohammed Jafri reports a relationship with AbbVie Inc that includes: speaking and lecture fees. Syed-Mohammed Jafri reports a relationship with Gilead Sciences Inc that includes: speaking and lecture fees. Syed-Mohammed Jafri reports a relationship with Takeda Pharmaceuticals USA Inc that includes: consulting or advisory and speaking and lecture fees. Syed-Mohammed Jafri reports a relationship with Ironwood Pharmaceuticals Inc that includes: consulting or advisory. Faruq Pradhan reports a relationship with Madrigal Pharmaceutical that includes: consulting or advisory. If there are other authors, they declare that they have no known competing financial interests or personal relationships that could have appeared to influence the work reported in this paper.
